# Miscellany

**Published:** 1887-07

**Authors:** 


### Miscellany.

Clairvoyante Diagnosis.—A physician in this city recently had under his care a case of typhoid fever. As the patient did not progress very rapidly toward recovery an officious relative consulted a notorious clairvoyante regarding the patient. She affected the usual

"trance state" and gave the following curious diagnosis which was copied by her daughter, who acts as amenuensis: "As I examine your case I find the membrain of the head has a watery fluid secreted in the membrain, which produces a sort of confusion and a roaring at the ears. The blood seems to loose its force of blood as it reaches the base of brain. this is caused by the strings of the kidneys drawing on the spinal nerves. this also is the cause of pain about the shoulder. I find both kidneys have been strained and the casings swell. I find the liver congested and filled with slime. this at times passes to the stomach, windpipe, left lung and throat. the left lung settles on casing of heart. I find a watery fluid between the heart and casing which causes the right ventrical and centre valve to be weak. the cough is caused by the left lung settling on the stomach. the stomach I find has been weakened by medicine, and I find a sort of white powder secreted in the casing of stomach and membrain of bowels, which is the effect of medicine. I find a little difficulty with bladder. I find a catching pain from hip to knee which has been caused from kidneys. I find the blood in a stagnant state, and the blood only quivers through the limbs.

"Take No. 5 with 10 Stomach Drops 5 times a day, and Tea of Sweet Balm and Poor Robin's Plantain."

As the clairvoyante had described only one symptom—the cough—in the case, the prescription was turned over to the attending physician.

---

THE CAUSATION OF MALARIAL FEVERS.—Dr. Jules Rouquette, in the *Bull. Gen. de Therapeutique*, claims that the poison-producing miasmatic diseases may be taken into the system either by the lungs or digestive tract. Pathological chemical actions are set up by the presence of the poison in the blood, but the fever will not develop unless the morbid leucomaines are formed more rapidly than they can be eliminated, when a high temperature is produced in the body to destroy them. In order that a locality may be "malarial," the following conditions must be fulfilled:

1. The air must come in contact with moist layers of soil.
2. There must be a temperature of at least 65° Fahrenheit.
3. There must be a persistent moisture of the soil.
4. There must be organic matter decomposing in the water or on soil recently covered by water.

5. There must be *beggiatoa* present in the decomposing mass. Beggiatoa are bacteria which live on the debris of organic matter, especially decomposing plants, and are most frequently found at the bottom of stagnant ponds. These bacteria decompose the sulphates of the water, liberating sulphuretted hydrogen gas.

---

LATENT GONORRHŒA.—Fritz Levy, in *Hospitals' Tidende*, publishes an elaborate article on this subject. The following are the conclusions of his paper:

1. The gonococcus of Neisser must be considered the specific cause of gonorrhœa.

2. The gonococcus is found both in chronic and acute gonorrhœa, and may be concealed in urethral discharges of many years standing.

3. The presence of the gonococcus in the secretion is intermittent.

4. The intensity of the gonorrhœal inflammation is not always proportionate with the number of gonococci.

5. In females the normal appearance of the vaginal and urethral mucosa is not a proof that the gonorrhœa is cured.

6. Uterine gonorrhœa is a dangerous affection in women, as the inflammation may extend through the Fallopian tubes to the ovary, and even into the peritoneal cavity. In these cases a contagious discharge is secreted at intervals for many years.

---

LOSS OF WEIGHT IN TYPHOID FEVER.—Dr. H. Cothin has made a careful study of the variations in the weight of the body in cases of typhoid fever, and has arrived at the following conclusions:

1. Typhoid fever presents two periods, a period of loss and a period of gain. Certain accidents and complications can modify these, but the general rule holds good.

2. The loss of weight is due more to the excessive combustion of tissues than to the defective diet.

3. The loss of nitrogen and of weight varies directly with the intensity of the fever.

4. A careful study of the variations in weight are of value in prognosis, an increase indicating convalescence.

5. Complications augment the loss of weight.

6. The study of weight variations enables us to determine the best diet for typhoid fever patients.

7. The loss of weight in individual cases progresses each day in a uniform manner.

---

Public exhibitions of hypnotism, magnetism, mind-reading, etc., have been prohibited in Switzerland, on the ground that they tend to encourage quackery and increase the superstition of the masses.

---

We have received from Dr. G. Apostoli, the eminent Parisian gynecologist, a monograph describing his new treatment of chronic metritis by means of the chemical galvano-cautery.

---

Nebraska has organized her first State Board of Health. The Board consists of seven physicians, of at least ten years' practice, the Governor being the appointing power.

---

Gilles de la Tourette reports, in *Annal Med. Leg.*, several cases in which young girls in the hypnotic state have been subject to criminal abuse.

---

It has been demonstrated by Galtier that the germs of bovine tubercles exist not only in the fresh milk, but are still found in condensed milk, and even in cheese.

---

A training school for male nurses has been established in connection with Bellevue Hospital by the Commissioners of Charity and Correction.

---

Last year there were 3,696 medical students in the University of Paris, 108 of whom were women.

